# Anti-adipogenic and Pro-lipolytic Effects on 3T3-L1 Preadipocytes by CX-4945, an Inhibitor of Casein Kinase 2

**DOI:** 10.3390/ijms23137274

**Published:** 2022-06-30

**Authors:** Anil Kumar Yadav, Byeong-Churl Jang

**Affiliations:** 1 Department of Molecular Medicine, College of Medicine, Keimyung University, 1095 Dalgubeoldaero, Dalseo-gu, Daegu 42601, Korea; yadav127@umn.edu; 2 The Hormel Institute, University of Minnesota, Austin, MN 55912, USA

**Keywords:** CX-4945, CK2, adipogenesis, lipolysis, 3T3-L1

## Abstract

Casein kinase 2 (CK2) is a ubiquitously expressed serine/threonine kinase and is upregulated in human obesity. CX-4945 (Silmitasertib) is a CK2 inhibitor with anti-cancerous and anti-adipogenic activities. However, the anti-adipogenic and pro-lipolytic effects and the mode of action of CX-4945 in (pre)adipocytes remain elusive. Here, we explored the effects of CX-4945 on adipogenesis and lipolysis in differentiating and differentiated 3T3-L1 cells, a murine preadipocyte cell line. CX-4945 at 15 μM strongly reduced lipid droplet (LD) accumulation and triglyceride (TG) content in differentiating 3T3-L1 cells, indicating the drug’s anti-adipogenic effect. Mechanistically, CX-4945 reduced the expression levels of CCAAT/enhancer-binding protein-α (C/EBP-α), peroxisome proliferator-activated receptor-γ (PPAR-γ), fatty acid synthase (FAS), acetyl-CoA carboxylase (ACC), and perilipin A in differentiating 3T3-L1 cells. Strikingly, CX-4945 further increased the phosphorylation levels of cAMP-activated protein kinase (AMPK) and liver kinase B-1 (LKB-1) while decreasing the intracellular ATP content in differentiating 3T3-L1 cells. In differentiated 3T3-L1 cells, CX-4945 had abilities to stimulate glycerol release and elevate the phosphorylation levels of hormone-sensitive lipase (HSL), pointing to the drug’s pro-lipolytic effect. In addition, CX-4945 induced the activation of extracellular signal-regulated kinase-1/2 (ERK-1/2), and PD98059, an inhibitor of ERK-1/2, attenuated the CX4945-induced glycerol release and HSL phosphorylation in differentiated 3T3-L1 cells, indicating the drug’s ERK-1/2-dependent lipolysis. In summary, this investigation shows that CX-4945 has strong anti-adipogenic and pro-lipolytic effects on differentiating and differentiated 3T3-L1 cells, mediated by control of the expression and phosphorylation levels of CK2, C/EBP-α, PPAR-γ, FAS, ACC, perilipin A, AMPK, LKB-1, ERK-1/2, and HSL.

## 1. Introduction

Obesity is a global pandemic responsible for the elevation of non-communicable diseases, including hyperlipidemia, type 2 diabetes, cancer, and cardiovascular disease, leading to comorbid issues and impaired health risks during the COVID-19 pandemic [1,2]. The prevalence of obesity is a global problem, with 13% of the adult population reported as being clinically obese [2]. It is mainly characterized by abnormal lipid accumulation as a result of adipocyte hyperplasia (significant rise in fat cell number) and hypertrophy (in fat cell size) [3]. A wealth of information illustrates that excessive preadipocyte differentiation causes abnormal accumulation and storage of high fat, mainly in triglyceride (TG), in mature adipocytes, which leads to the irregular development of the adipose tissue and the burgeoning of obesity [4]. Evidence also strongly indicates that numerous factors such as environmental and nutritional values and genetic and endocrine abnormalities are closely linked to the induction of obesity [5,6,7].

White adipose tissue (WAT) in the human body was considered only an energy storage organ. However, WAT is currently recognized as an endocrine organ that controls energy metabolism and homeostasis by secreting not only more than 600 bioactive factors known as adipokines but also different kinds of fatty acids and metabolites [8]. Adipokines largely influence adipocyte differentiation, metabolism, and function [9]. Preadipocyte differentiation, also known as adipogenesis, is a tightly controlled process in the form of morphological, biochemical, and cellular parameters [10]. This process transforms fibroblast-like preadipocytes into differentiated (or mature) adipocytes filled with numerous lipid droplets (LDs) [11,12]. There is accumulating evidence that diverse adipogenic transcription factors, including CCAAT/enhancer-binding proteins (C/EBPs), peroxisome proliferator-activated receptors (PPARs), and signal transducer and activator of transcription (STAT) family proteins, play a key role in preadipocyte differentiation [13,14,15].

Preadipocyte differentiation also involves lipogenesis and LDs maturation/stabilization, which requires fatty acid synthase (FAS), acetyl-CoA carboxylase (ACC), and perilipin A [16,17,18,19]. It also has been reported that several protein kinases, including protein kinase A (PKA), cAMP-activated protein kinase (AMPK), extracellular signal-regulated protein kinase-1/2 (ERK-1/2), p38 mitogen-activated protein kinase (MAPK), and protein kinase C (PKC), participate in the regulation of preadipocyte differentiation [17,20,21,22,23]. On the other hand, lipolysis is a process in which stored TG’s ester bonds in differentiated adipocytes is cleaved, resulting in the generation of free fatty acids and glycerol [24]. Hormone-sensitive lipase (HSL) is a master regulator in the mobilization of fatty acids from stored TG and is phosphorylated (activated) by PKA and ERK-1/2 in adipocytes [25]. Thus, any drug or molecular target that inhibits adipogenesis and promotes lipolysis in (pre)adipocytes could be a preventive and therapeutic option against obesity. 

Casein kinase 2 (CK2) is a ubiquitous and constitutively active serine (Ser, S)/threonine (Thr, T) kinase that is composed of two catalytic (α and/or α’) and two regulatory (β) subunits. CK2, which phosphorylates hundreds of substrates and controls several signaling pathways, is implicated in many human diseases [26]. Previously, it has been reported that CK2 plays a positive role during the adipocyte differentiation of 3T3-L1 cells and is induced in human obesity [27]. CX-4945 (Silmitasertib) is an inhibitor of CK2 with anti-cancerous and anti-angiogenic effects [28,29,30]. There is recent evidence showing that CX-4945 regulates the adipocyte differentiation of 3T3-L1 cells and human adipose tissue-derived stem cells [31], further pointing out the drug’s anti-adipogenic effect. 

However, the anti-adipogenic and pro-lipolytic effects and the mode of action of CX-4945 in (pre)adipocytes remain elusive. In this study, we investigated the regulatory effects and mechanisms of CX-4945 on lipid accumulation and lipolysis in differentiating and differentiated 3T3-L1 cells. We here demonstrate that CX-4945 has strong anti-adipogenic and pro-lipolytic effects on 3T3-L1 cells, and these effects are mediated through control of the expression and phosphorylation levels of CK2, C/EBP-α, PPAR-γ, FAS, ACC, perilipin A, AMPK, LKB-1, ERK-1/2, and HSL.

## 2. Results

### 2.1. CX-4945 Markedly Reduces Lipid Accumulation and TG Content in Differentiating 3T3-L1 Preadipocytes with no Significant Cytotoxicity 

**Figure 1:**
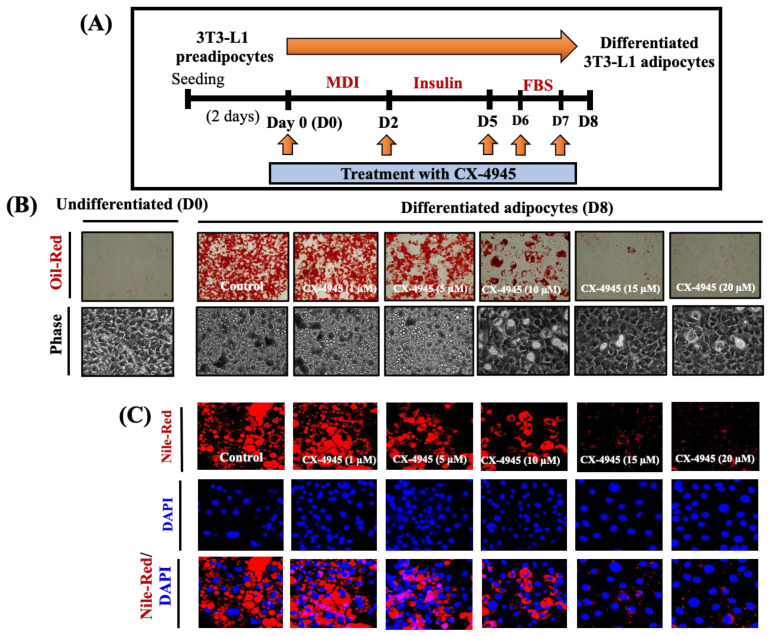

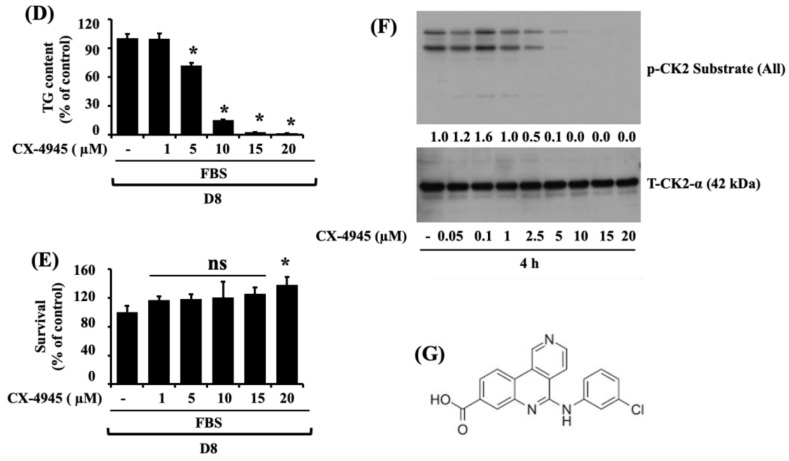
Effects of CX-4945, a CK2 kinase inhibitor, on lipid accumulation and cell survival during 3T3-L1 preadipocyte differentiation. (**A**) Scheme of 3T3-L1 preadipocyte differentiation. (**B**) 3T3-L1 preadipocytes were induced to differentiate with an induction medium containing MDI, insulin, and FBS in the presence or absence of different concentrations of CX-4945 up to D8 of differentiation. On D8, cellular lipid contents were assessed by Oil red O staining (upper panel) and phase-contrast images (lower panel). (**C**) After the above-mentioned condition, Nile red staining of the cells after the treatment. Data are mean ± SE (*n* = 3). * *p* < 0.05 vs. control (D8). (**D**) On D8, cellular TG content measurement was measured. (**E**) Cell survival assay was performed. Data are mean ± SE (*n* = 3). * *p* < 0.05 vs. control (D8). (**F**) 3T3-L1 preadipocytes were treated with different concentrations of CX-49 for 4 h. Whole-cell lysates were extracted and analyzed by immunoblot analysis with concern antibodies. (**G**) The chemical structure of CX-4945.

The experimental scheme and timescale for 3T3-L1 preadipocyte differentiation are shown in Figure 1A. During the differentiation process, cells were treated with or without CX4945 on D0, D2, D5, D6 and D7. Firstly, we underwent to determine the effects of CK2 inhibition by CX-4945 on fat storage and TG content during 3T3-L1 preadipocyte differentiation. As shown in Figure 1B, Oil red staining (upper panels), compared with the mock-treated 3T3-L1 cells, CX-4945 strongly inhibited lipid accumulation in a concentration-dependent manner in 3T3-L1 cells on D8 of differentiation. CX-4945 at 15 and 20 µM caused the maximal suppression of lipid accumulation. Furthermore, light microscopic observations (Figure 1B, lower panels) and Nile red staining (Figure 1C) confirmed the CX-4945’s lipid-decreasing effects. 

As shown in Figure 1D, CX-4945 also markedly reduced triglycerides (TG) content in a dose-dependent manner in 3T3-L1 cells on D8 of differentiation in which CX-4945 at 15 and 20 µM caused the maximal reduction of TG content. Of note, the results of cell count analysis demonstrated that CX-4945 at the dosages implemented was not toxic to 3T3-L1 cells at the time tested; rather, CX-4945 at 20 µM significantly (*p* < 0.05) enhanced the cell survival compared to the control (Figure 1E). As depicted in Figure 1F, treatment with CX-4945 decreased the phosphorylation of CK2 substrate in a concentration-dependent manner in 3T3-L1 preadipocytes, confirming the drug’s efficacy. The chemical structure of CX-4945 is shown in Figure 1G. Due to maximal suppressive impacts on lipid accumulation and TG content and inhibition of CK2 kinase activity without cytotoxicity, the 15 µM of CX-4945 was selected for further studies.

### 2.2. CX-4945 Decreases the Expression Levels of C/EBP-α and PPAR-γ during 3T3-L1 Preadipocyte Differentiation

**Figure 2:**
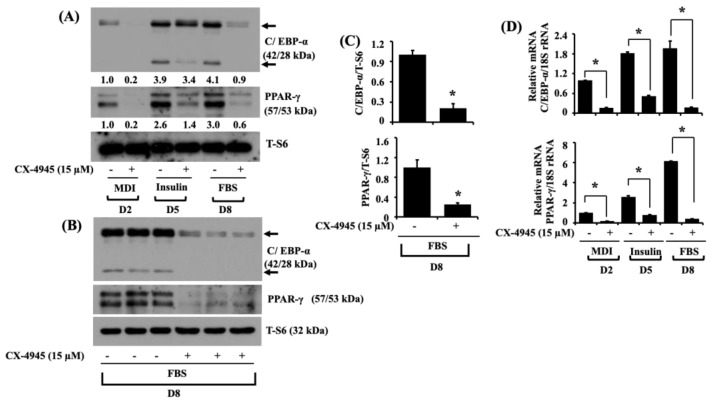
Effects of CX-4945 on the protein and mRNA expression levels of C/EBP-α and PPAR-γ during 3T3-L1 preadipocyte differentiation. (**A**) 3T3-L1 preadipocytes were induced to differentiate with an induction medium containing MDI, insulin, and FBS in the presence or absence of CX-4945 (15 µM) and harvested on D2, D5, and D8, respectively. Whole-cell lysates at each time point were extracted and analyzed by immunoblot analysis. (**B**) Immunoblot analysis in triplicate at D8. (**C**) Quantification of bands shown in (**B**). Data are mean ± SE (*n* = 3). * *p* < 0.05 vs. control on a respective day. (**D**) After the differentiation mentioned above, total mRNA at indicated time points was isolated and analyzed with real-time qPCR with respective primers. Data are mean ± SE (*n* = 3). * *p* < 0.05 vs. control on a respective day.

To delineate the molecular mechanisms by which CX-4945 causes less lipid accumulation and TG content during 3T3-L1 preadipocyte differentiation, we examined whether CX-4945 (15 µM) affects the expression levels of critical pro-adipogenic transcription factors, such as C/EBP-α and PPAR-γ in 3T3-L1 cells on D2, D5, and D8 of differentiation. As shown in Figure 2A, there was a decreased protein expression of C/EBP-α with high molecular weight (MW) on D2 or D8 and with low MW on D5 and D8 in CX4945-treated 3T3-L1 cells, compared with vehicle control (DMSO; 0.1%). Furthermore, CX-4945 strongly repressed the protein expression levels of PPAR-γ in 3T3-L1 cells on D2, D5, and D8. Total protein expression levels of a ribosomal protein S6, used as a loading control, was consistent under these experimental conditions. Triplicate experiments also affirmed the capability of CX-4945 to impede the protein expression levels of C/EBP-α and PPAR-γ in 3T3-L1 cells on D8 of differentiation (Figure 2B). The densitometry results of Figure 2B are exhibited in Figure 2C. Total expression levels of S6 protein remained unchanged under these experimental conditions. As shown in Figure 2D, the results of real-time qPCR analyses further demonstrated the reduced mRNA expression of C/EBP-α and PPAR-γ in CX-4945-treated 3T3-L1 cells, compared with vehicle control cells. 

### 2.3. CX-4945 Reduces the Expression Levels of FAS and Perilipin A during 3T3-L1 Preadipocyte Differentiation

**Figure 3:**
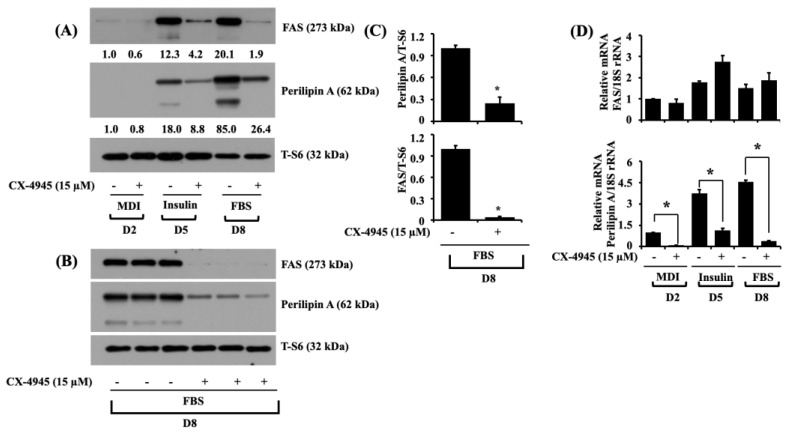
Effects of CX-4945 on the expression levels of FAS and perilipin A during 3T3-L1 preadipocyte differentiation. (**A**) 3T3-L1 preadipocytes were induced to differentiate with an induction medium containing MDI, insulin, and FBS in the presence or absence of CX-4945 (15 µM) and harvested on D2, D5, and D8, respectively. Whole-cell lysates at each time point were extracted and analyzed by immunoblot analysis. (**B**) Immunoblot analysis in triplicate at D8. (**C**) Quantification of bands in (**B**). Data are mean ± SE (*n* = 3). * *p* < 0.05 vs. control on a respective day. (**D**) After the differentiation mentioned above, total mRNA at the indicated time point was isolated and analyzed with real-time qPCR with respective primers. Data are mean ± SE (*n* = 3). * *p* < 0.05 vs. control on a respective day.

Next, we explored the effect of CX-4945 on the expression levels of FAS, an enzyme responsible for fatty acid synthesis [16], and perilipin A, an LD-binding and stabilizing protein [18,19], in 3T3-L1 cells on D2, D5, and D8 of differentiation. As shown in Figure 3A, CX-4945 (15 µM) significantly suppressed the protein expression levels of FAS and perilipin A on D5 and D8 of 3T3-L1 differentiation compared to vehicle control cells. Total expression levels of S6 protein remained constant under these experimental conditions. Triplicate experiments also affirmed the capability of CX-4945 to impede the expression levels of FAS and perilipin A in 3T3-L1 cells on D8 of differentiation (Figure 3B). The densitometry results of Figure 3B are exhibited in Figure 3C. Total protein expression levels of S6 remained unchanged under these experimental conditions. Moreover, as shown in Figure 3D, real-time qPCR analysis data, exhibited a significant reduction in the mRNA expression levels of perilipin A in the CX-4945-treated 3T3-L1 cells on D2, D5, and D8 of differentiation. However, the mRNA expression levels of FAS were unchanged or slightly increased during the differentiation of 3T3-L1 preadipocytes into adipocytes. 

### 2.4. CX-4945 Increases the Phosphorylation Levels of AMPK, LKB-1, and ACC While Decreasing the Intracellular ATP Content during 3T3-L1 Preadipocyte Differentiation

**Figure 4:**
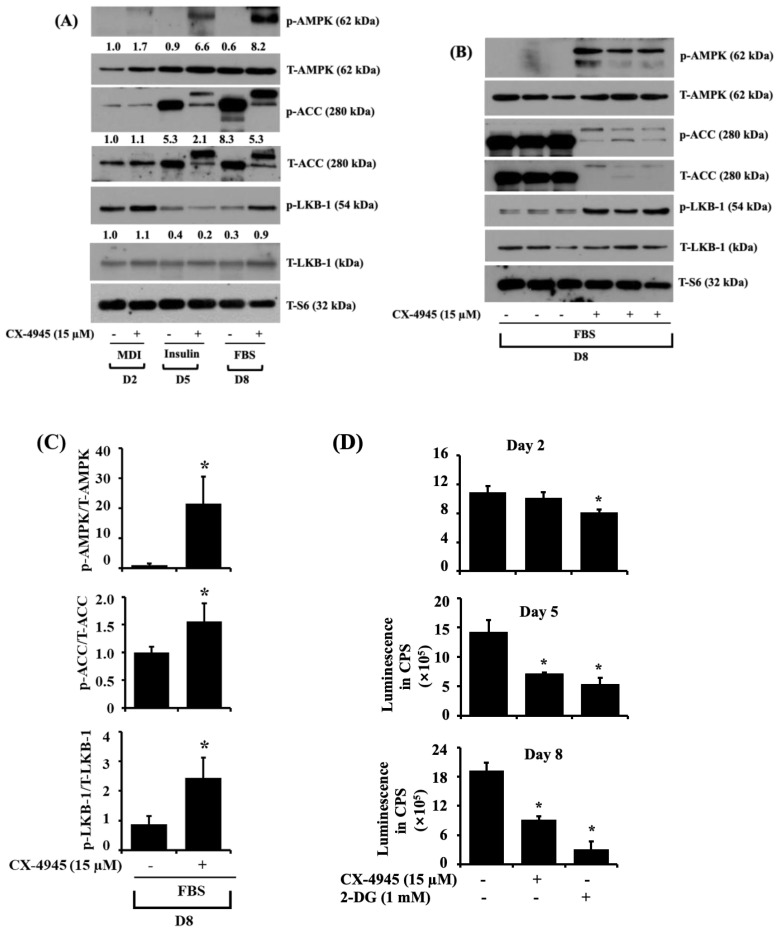
Effects of CX-4945 on the expression and phosphorylation levels of AMPK, ACC, LKB-1, and intracellular ATP content during 3T3-L1 preadipocyte differentiation. (**A**) 3T3-L1 preadipocytes were induced to differentiate with an induction medium containing MDI, insulin, and FBS in the presence or absence of CX-4945 (15 µM) and harvested on D2, D5, and D8, respectively. At each time point, whole-cell lysates were extracted and analyzed by immunoblot analysis. (**B**) Immunoblot analysis in triplicate at D8. (**C**) Quantification of bands in (**B**). Data are mean ± SE (*n* = 3). * *p* < 0.05 vs. control on a respective day. (**D**) 3T3-L1 preadipocytes were induced to differentiate with an induction medium containing MDI, insulin, and FBS in the absence or presence of CX-4945 or 2-deoxyglucose (2-DG), a known ATP depleting agent, and harvested at D2 (**A**), D5 (**B**), and D8 (**C**), respectively. The intracellular ATP content at the indicated time point was measured by an ATP measurement kit. Data are mean ± SE (*n* = 3). * *p* < 0.05 vs. control on a respective day.

AMPK is a crucial controller of energy metabolism, and its activation (phosphorylation on T172) leads to the inhibition of adipogenesis [20,32]. Thus, we next explored whether CX-4945 (15 µM) could modulate AMPK phosphorylation in differentiating 3T3-L1 cells. Notably, as exhibited in Figure 4A, CX-4945 strongly induced the phosphorylation of AMPK on D5 and D8 of 3T3-L1 differentiation compared to vehicle control. Total protein expression levels of AMPK remained constant under these experimental conditions. ACC is a well-known downstream effector of AMPK and is responsible for synthesizing fatty acids [33]. It should be noted that the nonphosphorylated ACC is active, but the phosphorylated one (on S79) is inactive. Herein, of note, there was a substantial increase in phosphorylation (a dotted arrow) and total expression (an arrow) levels of ACC in 3T3-L1 cells on D5 and D8 of differentiation. Strikingly, treatment with CX-4945 decreased the total expression levels of ACC while increasing another phosphorylation and total expression levels of ACC with higher molecular mass in 3T3-L1 cells on D5 and D8 of differentiation. Considering that liver kinase B-1 (LKB-1) is a crucial kinase to induce the phosphorylation (T172) of AMPK [34], we further determined whether LKB-1 protein is expressed and activated (phosphorylated on S428) in differentiating 3T3-L1 cells and CX-4945 regulate it. Interestingly, compared with vehicle control, CX-4945 treatment enhanced LKB-1 phosphorylation levels in 3T3-L1 cells on D2 and D8 of differentiation. Total protein expression levels of LKB-1 remained essentially unchanged under these experimental conditions. As exhibited in Figure 4B, the results of triplicate experiments also affirmed the capability of CX-4945 to induce the phosphorylation and expression levels of AMPK, ACC, and LKB-1 in 3T3-L1 cells on D8 of differentiation. The densitometry results of Figure 4B are exhibited in Figure 4C.

The phosphorylation (activation) of AMPK is affected by variations in the intracellular AMP/ATP ratio [35]. This promptly led us to investigate whether CX-4945 affects intracellular ATP levels during 3T3-L preadipocytes differentiation. As depicted in Figure 4D, treatment of CX-4945 led to a significant reduction of the intracellular ATP content in 3T3-L1 cells on D2 and D8 of differentiation compared to vehicle control. Similarly, as a positive control used, 2-deoxyglucose (2-DG) lowered the intracellular ATP content in 3T3-L1 cells for the times tested.

### 2.5. CX-4945 Down-Regulates the mRNA Expression Levels of Leptin and Resistin during 3T3-L1 Preadipocyte Differentiation 

**Figure 5:**
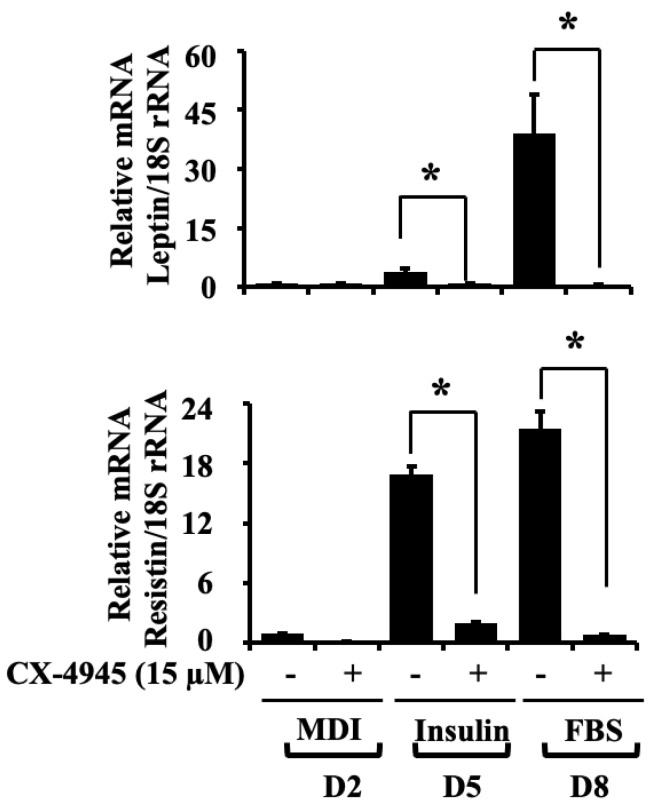
Effects of CX-4945 on the mRNA expression levels of leptin and resistin during 3T3-L1 preadipocyte differentiation. 3T3-L1 preadipocytes were induced to differentiate with an induction medium containing MDI, insulin, and FBS in the presence or absence of CX-4945 (15 µM) and harvested on D2, D5, and D8, respectively. Total RNA at each time point was extracted and analyzed by real-time qPCR with respective primers. Data are mean ± SE (*n* = 3). * *p* < 0.05 vs. control on a respective day.

Adipokines, including leptin and resistin, are mainly secreted from adipose tissue and adipocytes. Recent studies have indicated the vital aspect of adipokines in obesity and related diseases [9]. Herein, we determined the mRNA expression levels of leptin and resistin after the treatment with CX-4945 during 3T3-L1 preadipocytes differentiation. As exhibited in Figure 5, CX-4945 vastly decreased the mRNA expression levels of leptin and resistin in 3T3-L1 cells on D5 and D8 of differentiation compared with vehicle control. 

### 2.6. CX-4945 Can Increase Glycerol Release and the Phosphorylation Levels of HSL in Differentiated 3T3-L1 Adipocytes

**Figure 6:**
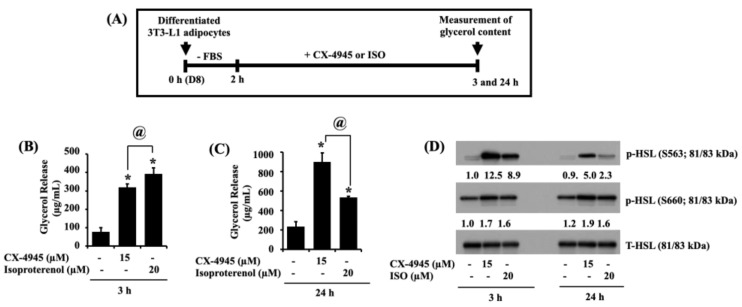
Effects of CX-4945 on glycerol release and the expression and phosphorylation levels of HSL in differentiated 3T3-L1 adipocytes. (**A**) The experimental scheme for measuring glycerol content and HSL phosphorylation in differentiated 3T3-L1 cells. (**B**,**C**) Differentiated 3T3-L1 cells were serum-starved for 2 h and treated with CX-4945 or ISO for the indicated doses and time points. Glycerol content was measured at 3 h (**B**) and 24 h (**C**). Data are mean ± SE (*n* = 3). * *p* < 0.05 vs. control (3 or 24 h). (**D**) After the treatment mentioned above, whole-cell lysates were extracted and analyzed by immunoblot analysis with respective antibodies.

Next, we determined whether CX-4945 has a lipolytic effect on differentiated 3T3-L1 adipocytes. Herein, the CX-4945’s lipolytic effect was evaluated by its ability to stimulate glycerol content in the culture medium and the intracellular phosphorylation levels of HSL (on S563 and S660) from the conditioned cells. As a positive control, isoproterenol (ISO) was used [36]. The experimental scheme and timescale for the measurement of glycerol content and HSL phosphorylation are depicted in Figure 6A. As shown in Figure 6B,C, treatment with ISO for 3 and 24 h significantly increased glycerol release in differentiated 3T3-L1 adipocytes. Of note, CX-4945 treatment at 3 and 24 h also substantially increased glycerol release in these cells. Furthermore, as expected, ISO greatly enhanced HSL phosphorylation on S563 and S660 without altering its total protein expression levels in differentiated 3T3-L1 adipocytes (Figure 6D). Of interest, CX-4945 treatment at times tested further vastly increased HSL phosphorylation on S563 and S660 with no alteration of the protein total expression levels in differentiated 3T3-L1 adipocytes.

### 2.7. CX-4945 INDUCES the Phosphorylation of ERK-1/2 and Perilipin A, but Not PKA, and PD98059, an Inhibitor of ERK-1/2, Powerfully Block the CX-4945-Induced Glycerol Release and Phosphorylation of HSL and Perilipin A in Differentiated 3T3-L1 Adipocytes 

**Figure 7:**
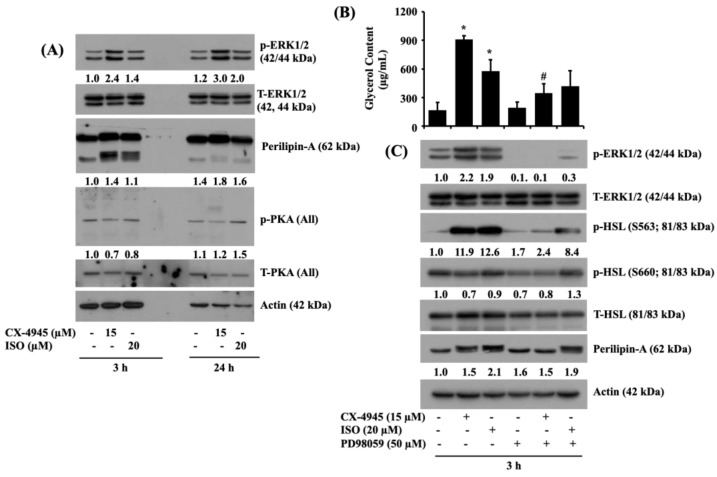
Effect of PD98059, an inhibitor of ERK-1/2, on CX-4945-induced lipolysis in differentiated 3T3-L1 cells. (**A**) Differentiated 3T3-L1 cells were serum-starved for 2 h and treated with CX-4945 or ISO for indicated time points. After-above mentioned treatment conditions, the cellular protein was extracted and analyzed by Western blot analysis with respective antibodies. (**B**) Differentiated 3T3-L1 cells were serum-starved for 2 h and treated with CX-4945 or ISO in the presence or absence of PD98059 for 3 h. Glycerol content was measured at an indicated time point. Data are mean ± SE (*n* = 3). * *p* < 0.05 vs. control (no drug). # *p* < 0.05 vs. CX-4945 (15 μM). (**C**) After the treatment mentioned above, whole-cell lysates were extracted and analyzed by immunoblot analysis with respective antibodies.

Evidence suggests that the activation of ERK-1/2, perilipin A, and protein kinase A (PKA) regulates lipolysis [24,25]. We thus investigated whether CX-4945 modulates the phosphorylation and expression levels of ERK-1/2, perilipin A, and PKA in differentiated 3T3-L1 cells. As expected, treatment with ISO at 3 and 24 h led to a slight increase in the phosphorylation levels of ERK-1/2 and PKA in differentiated 3T3-L1 cells (Figure 7A). ISO treatment at 3 h (but not 24 h) further induced an electrophoretic shift (hyperphosphorylation) of the expression of perilipin A in differentiated 3T3-L1 cells. Strikingly, treatment with CX-4945 at 3 and 24 h led to stronger induction of the phosphorylation levels of ERK-1/2 and perilipin A in differentiated 3T3-L1 cells than those in the ISO-treated cells. Distinctly, CX-4945 treatment at 3 and 24 h had fewer phosphorylation levels of PKA in differentiated 3T3-L1 cells than those in the ISO-treated cells. Total protein expression levels of ERK-1/2 and PKA and loading control actin remain unchanged under these experimental conditions. To evaluate the role of ERK-1/2 activation in the CX-4945-induced lipolysis, we next tested the effects of PD98059, a specific MEK1/2 inhibitor, on the CX-4945 or ISO-induced glycerol release and HSL phosphorylation in differentiated 3T3-L1 cells. As shown in Figure 7B, CX-4945 or ISO treatment for 3 h led to increased glycerol content in differentiated 3T3-L1 cells. Of note, while PD98059 treatment partially blocked the CX-4945-induced glycerol release in differentiated 3T3-L1 cells, this MEK-1/2 (ERK-1/2) inhibitor significantly blocked it. As exhibited in Figure 7C, treatment of PD98059 that strongly reduced both basal and the CX-4945-induced ERK-1/2 phosphorylation without changing its total protein expression levels vastly interfered with the CX4945-induced phosphorylation of HSL and electrophoretic shift (hyperphosphorylation) of perilipin A in differentiated 3T3-L1 cells. In contrast, PD98059 had more minor inhibitory effects on the ISO-induced phosphorylation of ERK-1/2, HSL, and perilipin A in differentiated 3T3-L1 cells. Total protein expression levels of ERK-1/2 and PKA and loading control actin remain unchanged under these experimental conditions.

## 3. Discussion

CK2 is a ubiquitously expressed serine/threonine kinase with many protein substrates and plays a pivotal role in cell proliferation, survival, differentiation, and metabolism. CK2 is emerging as a mediator for adipocyte differentiation. However, the expression and function of CK2 in adipocyte differentiation and lipolysis are not fully understood. CX-4945 is a CK2 inhibitor known for its anti-cancerous and anti-adipogenic activities. The anti-adipogenic and lipolytic effects and mechanisms of CX-4945 also remain elusive. In this study, we investigated whether CK2 is expressed (and activated), and CX-4945 inhibits adipogenesis and promotes lipolysis in differentiating and differentiated 3T3-L1 cells. Here we demonstrate firstly that CK2 is expressed and CX-4945 inhibits lipid accumulation (adipogenesis) in differentiating 3T3-L1 preadipocytes, and the drug also promotes glycerol release (lipolysis) in differentiated 3T3- L1 cells via control of the expression and phosphorylation levels of CK2, C/EBP-α, PPAR-γ, FAS, perilipin A, AMPK, ACC, ERK-1/2, and HSL. 

Reportedly, the expression levels of CK2 are elevated during the adipocyte differentiation of 3T3-L1 cells [27,37]. We herein have demonstrated that CK2 is substantially expressed, and the phosphorylated CK2 substrates are also highly detected in 3T3-L1 preadipocytes. These results indicate that the expressed CK2 is functional in 3T3-L1 preadipocytes. The present study has further revealed that treatment with CX-4945 results in a concentration-dependent reduction of the phosphorylated levels of CK2 substrates in 3T3-L1 preadipocytes without altering the total protein expression levels of CK2, pointing out the drug’s efficacy to only inhibit CK2 activity. Importantly, data of Oil-red and Nile-red staining and TG measurement assay in this study further showed that CX-4945 dose-dependently inhibited lipid accumulation and TG content in 3T3-L1 cells on D8 of differentiation with no cytotoxicity. These results collectively suggest that CX-4945 suppresses lipid accumulation (adipogenesis) and TG synthesis (lipogenesis) in differentiating 3T3-L1 preadipocytes, likely mediated by inhibiting CK2 activity. 

A wealth of information illustrates the pivotal roles of C/EBP-α and PPAR-γ transcription factors in 3T3-L1 preadipocyte differentiation [14,15,34,37]. Both play as master transcriptional regulators of preadipocytes ‘entire terminal differentiation process. The CX-4945 regulation of C/EBP-α and PPAR-γ during 3T3-L1 preadipocyte differentiation is largely unknown. Considering the present findings that CX-4945 vastly impinges the expression levels of C/EBP-α and PPAR-γ during the adipocyte differentiation of 3T3-L1 cells, it is conceivable that the drug’s anti-adipogenic effect is further attributable to the reduced expression of these transcription factors. It is also documented that the expressions of FAS and perilipin A are required for adipocyte differentiation and maturation [9,16,18,19]. FAS is a lipogenic enzyme responsible for synthesizing fatty acids [16], and perilipin A is an LD-binding and stabilizing protein during preadipocyte differentiation [18,19]. Thus, given that CX-4945 suppresses the expression of both FAS and perilipin A in differentiating 3T3-L1 preadipocytes, it is speculative that the drug’s anti-lipogenic and lipid-lowering effects are further linked to the downregulation of FAS and perilipin A. It is known that adipokines, including adiponectin, leptin, and resistin, are mainly secreted from adipose tissue and adipocytes. Reportedly, adiponectin expression decreases with an increase in adiposity [38], but levels of leptin and resistin expression increase in obesity [39]. It is also postulated that resistin is linked to obesity, insulin resistance, and diabetes [40]. Consequently, suppression of leptin and resistin expressions is an alternative against obesity. Considering the capability of CX-4945 to decrease the expression levels of pathogenic leptin and resistin during 3T3-L1 preadipocyte differentiation, it is suggested that CX-4945 may be used as a potential anti-obesity drug candidate or hit to target obesity and related diseases in which overexpression of leptin and resistin is problematic.

AMPK is the master regulator of energy metabolism and balance [20,32]. It is a heterotrimeric protein kinase consisting of a catalytic α subunit, regulatory β and γ subunits. Current research has shown that an elevation of intracellular AMP/ATP ratio induces AMPK phosphorylation (activation) on T172 [35]. It also has been reported that AMPK is phosphorylated by specific upstream protein kinases, including LKB-1 [35]. Accordingly, the phosphorylation (activation) of AMPK inhibits ATP-consuming anabolic processes while activating ATP-producing catabolic processes [41,42], which are mediated through the AMPK-dependent phosphorylation of metabolic enzymes, such as ACC [43]. There is also evidence that AMPK activation induces ACC phosphorylation on S79, which strictly regulates the enzyme during fatty acid synthesis for malonyl-CoA production, and phosphorylated ACC lacks its enzymatic activity to synthesize fatty acids [44]. The CK2 and CX-4945 regulation of phosphorylation and expression of AMPK, ACC, and LKB-1 during 3T3-L1 preadipocyte differentiation is unknown. In the current study, we have demonstrated that CX-4945 vastly increases the phosphorylation and expression of AMPK and ACC in differentiating 3T3-L1 preadipocytes. These results thus indicate that the CX-4945’s anti-adipogenic and anti-lipogenic effects are also mediated through the activation of AMPK and inhibition of ACC, which may confer inhibition of ATP-consuming anabolic processes, such as the synthesis of fatty acids herein. Furthermore, the present study has shown that CX-4945 increases LKB-1 phosphorylation while lowering cellular ATP content in differentiating 3T3-L1 preadipocytes. The CX-4945-induced AMPK phosphorylation during the adipocyte differentiation of 3T3-L1 cells is likely to be due to LKB-1 activation and the reduction of intracellular ATP content. 

Lipolysis involves the hydrolytic cleavage of ester bonds in stored TG in differentiated adipocytes, resulting in the release of free fatty acids and glycerol as a byproduct. HSL is a pivotal lipolytic enzyme that involves mobilizing fatty acids from stored TG in differentiated adipocytes [24]. It is also worthy to state previous studies that perilipin A is phosphorylated and proteolytically degraded in differentiated adipocytes exposed to specific lipolytic inducers such as β3-adrenergic receptor agonists (CL315263 or norepinephrine), which facilitate access to HSL to LDs to breakdown [44]. Accumulating evidence indicates that activation of several protein kinases, such as protein kinase A (PKA) and extracellular signal-regulated kinase-1/2 (ERK-1/2), induces lipolysis by phosphorylating HSL on S563 and S660 and perilipin A in differentiated adipocytes [45,46]. The CX-4945 regulation of lipolysis and the phosphorylation of ERK-1/2, PKA, HSL, and perilipin A in differentiated 3T3-L1 cells is unknown. Herein, considering the CX-4945’s capability to elevate glycerol release and the phosphorylation of HSL at S563 and S659 and perilipin A, it is obvious that the drug has a lipolytic effect on differentiated 3T3-L1 cells. Notably, the present study has further shown that CX-4945 selectively induces the activation of ERK-1/2 in differentiated 3T3-L1 cells, and PD98059, an ERK-1/2 inhibitor, powerfully inhibits not only the CX-4945-induced ERK-1/2 activation but also the drug-induced glycerol release and phosphorylation of HSL and perilipin A in these cells. These results indicate that CX-4945 appears to exert its lipolytic effect via the ERK-1/2-dependent HSL activation and perilipin A phosphorylation (degradation) in differentiated adipocytes.

**Figure 8:**
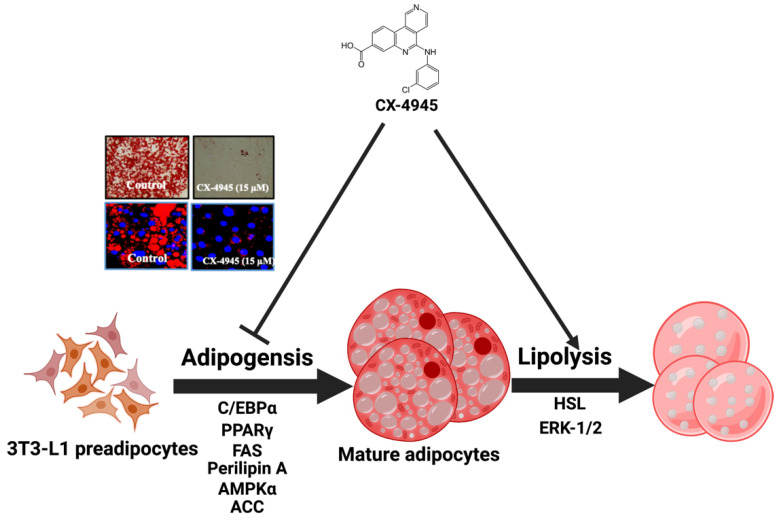
A graphical abstract showing the CX-4945’s anti-adipogenesis and pro-lipolytic mechanisms in differentiating and differentiated 3T3-L1 cells (Created with BioRender.com).

It is suggested that a likely scenario of the molecular and cellular mechanisms underlying the CX-4945’s anti-adipogenic and pro-lipolytic effects on 3T3-L1 cells herein is that (1) CX-4945 reduces the expression of adipogenesis-related C/EBP-α and PPAR-γ in differentiating 3T3-L1 cells, 2) CX-4945 lowers the expression of lipogenesis-related FAS and ACC in differentiating 3T3-L1 cells, 3) CX-4945 down-regulates the expression of perilipin A that binds and stabilizes nascent LDs in differentiating 3T3-L1 cells, 4) CX-4945 elevates the activation (phosphorylation) of AMPK and LKB-1 while lowering intracellular ATP levels in differentiating 3T3-L1 cells, 5) CX-4945 induces the ERK-1/2-dependent HSL activation (phosphorylation) in differentiated 3T3-L1 cells (Figure 8).

In summary, our results demonstrate for the first time that CX-4945 has strong anti-adipogenic and lipolytic effects on differentiating and differentiated 3T3-L1 cells, and these effects are mediated through control of the expression and phosphorylation levels of CK2, C/EBP-α, PPAR-γ, FAS, ACC, perilipin A, AMPK, LKB-1, ERK-1/2, and HSL. Even if essential questions such as anti-adipogenic and lipolytic effects of CX-4945 on obese animal models remain to be resolved, the present findings advocate CX-4945 as a potential therapeutic for treating obesity.

## 4. Materials and Methods

### 4.1. Drugs and Antibodies

CX-4945 was purchased from Selleckchem (Houston, TX, USA). 3-isobutyl-1-methylxanthine (IBMX), dexamethasone, and insulin were bought from Sigma (St. Louis, MO, USA). A detailed list of antibodies used in this study is included in Supplementary Table S1.

### 4.2. Cell Culture

Murine 3T3-L1 preadipocytes (CL-173TM, ATCC, Manassas, VA, USA) were grown in DMEM (high glucose) complemented by 10% heat-inactivated fetal calf serum (FCS, Gibco, Gaithersburg, MD, USA) and 1% penicillin-streptomycin cocktail at 37 °C in a 5% CO2 incubator.

### 4.3. Differentiation of 3T3-L1 Preadipocytes

Murine 3T3-L1 white preadipocytes were differentiated according to our previously reported method [47]. Briefly, cells were seeded in DMEM containing 10% fetal calf serum and penicillin/streptomycin mixture and maintained up to the contact inhibition stage for 2 days. Differentiation of 3T3-L1 was stimulated by adding induction media containing 10% FBS along with mixtures of hormones (MDI) such as 0.5 mM IBMX (M), 0.5 µM dexamethasone (D), and 5 µg/mL insulin (I) in the presence or absence of CX-4945. After 48 h of MDI-induction, DMEM supplemented with 10% FBS and 5 µg/mL insulin was replaced for the next 3 days with or without CX-4945. After a day (D) 5, cells were nourished every day with DMEM supplemented with 10% FBS until D8 in the presence or absence of CX-4945. 

### 4.4. Oil Red O Staining

On day eight of differentiation, conditioned 3T3-L1 were rinsed with phosphate-buffered saline (PBS) and followed fixation with 10% formaldehyde at room temperature (RT). Later, fixed cells were washed with 60% isopropanol and dried. A working solution of Oil red O (Sigma; St. Louis, MO, USA) was layered onto the cells. After 1 h, stained cells were washed with distilled water, and stored intracellular lipid droplets were visualized using an inverted microscope (Nikon, Tokyo, Japan). 

### 4.5. Nile-Red Staining

On day eight of differentiation, formalin-fixed cells were washed twice with distilled water, followed by washing with 60% isopropanol and cells were dried. Then, cells were incubated with Nile Red (1 µM in PBS) for 10 min at RT in dark conditions. After 3 extensive washes with PBS, cells were treated with 4′,6-diamidino-2-phenylindole dihydrochloride (DAPI; 100 ng/ml in PBS) as a nuclear counterstain (5 min; RT; dark). The images of stained lipid droplets were taken using a confocal microscope (Carl Zeiss GmbH, Vienna, Austria) 

### 4.6. Cell Survival Assay

3T3-L1 preadipocytes were seeded in a 24-well plate and grown under the above-mentioned differentiation conditions in the presence or absence of indicated concentrations of CX-4945. On D8 of differentiation, control or CX-4945-treated 3T3-L1 cells, which cannot be stained with 0.4 % trypan blue dye were counted under the optical microscope, respectively. The cell count analysis was carried out in triplicates. Data are mean ± standard error (SE) of three independent experiments [48].

### 4.7. Measurement of Intracellular TG

On day eight of differentiation, intracellular TG content in control (DMSO; 0.1%) or CX-4945-treated 3T3-L1 cells was quantified using the AdipoRed reagent kit (Lonza, Basel, Switzerland) as previously reported [49]. Fluorescence intensity was quantified with excitation and emission at 485 and 572 nm, respectively, using Victor3 (Perkin Elmer, Shelton, CT, USA).

### 4.8. Quantification of Glycerol Content

On D8, fat accumulated 3T3-L1 adipocytes were placed in serum-free media for 2 h and later incubated with CX-4945 (15 µM) or isoproterenol (ISO, 20 µM) for an additional 3 h and 24 h, respectively. At designated periods, the culture medium was saved, and glycerol content was quantified using free glycerol reagent (Sigma, St. Louis, MO, USA) as per the manufacturer’s instructions.

### 4.9. Preparation of Whole-Cell Lysates

At the designated time points, 3T3-L1 cells were washed with PBS and homogenized in a modified RIPA buffer (Sigma, St. Louis, MO, USA) containing a proteinase inhibitor cocktail. Later, lysates were collected by centrifugation at 12,074× *g* for 20 min at 4 °C. Protein concentration was evaluated by bicinchoninic acid (BCA) protein assay kit (Thermo Scientific, Rockford, IL, USA) [49].

### 4.10. Immunoblot Analysis

Protein samples (50 µg) were separated using 10% SDS-polyacrylamide gel electrophoresis (SDS-PAGE) and transferred onto polyvinylidene difluoride membrane (PVDF, Millipore, Bedford, MA, USA) by electroplating. Membranes were blocked with 5% skim milk prepared in TBST (1×) and subsequently incubated overnight with specific antibodies listed in Table S1 at 4 °C. Later, membranes were washed three times with TBST (1×) followed by incubation with horseradish peroxidase-conjugated secondary antibodies for 2 h at RT. Next, immunoblots were rinsed three times with TBST (1×) and protein bands were detected using enhanced chemiluminescence (ECL) reagents (Advansta, San Jose, CA, USA). Actin and total S6 proteins were used as an equal protein loading control [50].

### 4.11. Quantitative Real-Time PCR

Total RNA was obtained from control or CX-4945-treated 3T3-L1 cells using RNAiso Plus (TaKaRa, Kusatsu, Shiga, Japan). The isolated RNA (3 µg) was converted to cDNA using a random hexadeoxynucleotide primer and reverse transcriptase. For qPCR, SYBR green (TaKaRa, Kusatsu, Shiga, Japan) was used to determine transcript levels of genes with the LightCyclerâ96 Machine (Roche, Mannheim, Germany). PCR reactions were run in triplicate for each sample, and transcript levels of each gene were normalized to the level of 18S rRNA [34]. Primer sequences used in this study are listed in Table S2.

### 4.12. Quantification of Intracellular ATP Content

Intracellular ATP content during the differentiation of 3T3-L1 preadipocytes into adipocytes in the presence or absence of CX-4945 or 2-deoxyglucose (2-DG) was measured with ATPLite-1step kit from PerkinElmer (Shelton, CT, USA) according to the manufacturer’s instructions. 

### 4.13. Statistical Analyses

Cell count analysis was carried out in triplicate and repeated three times. Data are expressed as mean ± standard error (SE) of the mean. One-way ANOVA followed by Dunnett’s post hoc test or two-tailed Student’s t-test was performed using SPSS 11.5 software (SPSS, Inc., Chicago, IL, USA). *p* < 0.05 was considered to indicate statistically significant differences.

## Data Availability

Data is contained within the article.

## Supplementary Materials

The following supporting information can be downloaded at: https://www.mdpi.com/article/10.3390/ijms23137274/s1.
