# Supplementary material for: Anti-adipogenic and Pro-lipolytic Effects on 3T3-L1 Preadipocytes by CX-4945, an Inhibitor of Casein Kinase 2
Source: Int J Mol Sci. 2022 Jun 30;23(13):7274. doi: 10.3390/ijms23137274 (PMC9266649; doi:10.3390/ijms23137274)
Supplement: Supplementary file 1 [file ijms-23-07274-s001.zip › ijms-1774823-supplementary.pdf]

Table S1: List of antibodies used for Western blot analysis.

Antibodies	Dilution used	Source	Catalog no.
Primary antibodies			
C/EBP-α	1:2,000	Santa Cruz Biotechnology	sc-61
PPAR-γ	1:2,000	Santa Cruz Biotechnology	sc-7272
Perilipin A	1:2,000	BioVision	#3948-200
FAS	1:2,000	BD Bioscience	#9452
p-AMPK (T172)	1:2,000	Cell signalling	#2535
AMPK	1:2,000	Cell signalling	#2793
p-ACC (S79)	1:2,000	Cell signalling	#3661
ACC	1:2,000	Cell signalling	#3662
p-LKB-1 (S428)	1:2,000	Cell signalling	#3482
LKB-1	1:2,000	Cell signalling	#3047
p-HSL (S563)	1:2,000	Cell signalling	#4139
p-HSL (S660)	1:2,000	Cell signalling	#4126
HSL	1:2,000	Cayman chemical	#10006371
p-ERK1/2 (Thr202/Tyr204	1:2,000	Cell signalling	#9101
ERK1/2	1:2,000	Cell signalling	#4695
p-PKA	1:2,000	Santa Cruz Biotechnology	sc-377575
PKA	1:2,000	Santa Cruz Biotechnology	sc-365615
Phospho-CK2 Substrate [(pS/pT)DXE]	1:2,000	Santa Cruz Biotechnology	#8738
CK-2α	1:2,000	Santa Cruz Biotechnology	#2656
S6	1:5,000	Cell signalling	#2217
Secondary antibodies			
Goat anti-rabbit IgG-HRP	1:5000	Jackson ImmunoResearch	111-035-045
Goat anti-mouse IgG-HRP	1:5000	Jackson ImmunoResearch	115-035-062

Table S2: Sequences of primers used for quantitative real-time PC

Gene	Forward	Reverse
C/EBP-α	TTACAACAGGCCAGGTTTCC	CTCTGGGATGGATCGATTGT
PPAR-γ	GGTGAAACTCTGGGAGATTC	CAACCATTGGGTCAGCTCTC
FAS	TTGCTGGCACTACAGAATGC	AACAGCCTCAGAGCGACAAT
Perilipin A	CTTTCTCGACACACCATGGAAAC	CCACGTTATCCGTAACACCCTTCA
Leptin	CCAAAACCCTCATCAAGACC	CTCAAAGCCACCACCTCTGT
Resistin	CCGATGAGCAGTCA CCTCCA	CAGCTGCTTCGCCTCGTCCTCCT
18S rRNA	GGTGAAGGTCGGTGTGAACG	GGTAGGAACACGGAAGGCCA
